# Identification of separation-related problems in domestic cats: A questionnaire survey

**DOI:** 10.1371/journal.pone.0230999

**Published:** 2020-04-15

**Authors:** Daiana de Souza Machado, Paula Mazza Barbosa Oliveira, Juliana Clemente Machado, Maria Camila Ceballos, Aline Cristina Sant’Anna

**Affiliations:** 1 Programa de Pós-Graduação em Comportamento e Biologia Animal, Universidade Federal de Juiz de Fora, Juiz de Fora, Minas Gerais, Brazil; 2 Núcleo de Estudos em Etologia e Bem-estar Animal, Universidade Federal de Juiz de Fora, Juiz de Fora, Minas Gerais, Brazil; 3 Centro Universitário do Sudeste Mineiro (UNICSUM), Juiz de Fora, Minas Gerais, Brazil; 4 Swine Teaching and Research Center, Department of Clinical Studies, New Bolton Center, School of Veterinary Medicine, University of Pennsylvania, PA, United States of America; 5 Departamento de Zoologia, Universidade Federal de Juiz de Fora, Juiz de Fora, Minas Gerais, Brazil; Memorial University of Newfoundland, CANADA

## Abstract

Identifying and preventing the occurrence of separation-related problems (SRP) in companion animals are relevant to animal welfare and the quality of human-pet interactions. The SRP are defined as a set of behaviors and physiological signs displayed by the animal when separated from its attachment person. In cats, SRP has been insufficiently studied. Thus, the objective of this study was to develop a questionnaire for cat owners which identifies behaviors that may indicate SRP, as well as relates the occurrence of SRP to the management practices applied in the sampled cats. The associations of SRP with cats’ characteristics, as well as owner, environmental, and management traits were investigated. The questionnaire was developed based on the scientific literature about separation anxiety syndrome in dogs and a few papers in cats, and it was completed by 130 owners of 223 cats. Analysis of owners’ answers was done through categorization and acquisition of relative frequencies of each response category, followed by Fisher’s exact test, chi-square tests in contingency table and Multiple Correspondence Analysis. Among the sampled animals, 13.45% (30 / 223) met at least one of the behavioral criteria we used to define SRP. Destructive behavior was the most frequently reported behavior (66.67%, 20 / 30), followed by excessive vocalization (63.33%, 19 / 30), urination in inappropriate places (60.00%, 18 / 30), depression-apathy (53.33%, 16 / 30), aggressiveness (36.67%, 11 / 30) and agitation-anxiety (36.67%, 11 / 30) and, in lower frequency, defecation in inappropriate places (23.33%, 7 / 30). The occurrence of SRP was associated with the number of females living in the residence (P = 0.01), with not having access to toys (P = 0.04), and no other animal residing in the house (P = 0.04). Separation-related problems in domestic cats are difficult to identify due to the limited amount of knowledge regarding the issue. The questionnaire developed in this study supported identification of the main behaviors likely related to SRP in cats and could be used as a starting point for future research.

## Introduction

Behavioral problems in companion animals are among the main causes of abandonment in many countries, such as the United States of America, Japan and the United Kingdom [1–4]. For cats, the abandonment usually occurs when the animal exhibits behaviors perceived by owners as problematic, such as aggressiveness towards people and other animals in the house, inappropriate elimination and destructive behavior directed at the house [1, 2, 5–7]. Other behaviors considered problematic but natural to cats include scratching, climbing to high places, nocturnal activities, attention seeking, plant chewing, attempts to escape from the home and vocalizations [8, 9].

As the cat gains greater popularity as a companion animal [10, 11], there is increasing need for knowledge about the human-cat relationship and how it affects cats’ behavior and welfare [12, 13]. There is a belief that cats can easily cope with the owners’ absence for long periods of time and few studies have been conducted to support that assumption [14]. Recent studies have reported that cats can be considered as social, being able to generate bonds with their owners, and therefore it is likely they also show behaviors and physiological reactions due to the owners’ absence [15–19]. For instance, an experiment conducted to verify the attachment of cats towards their owners, using a modified version of the Ainsworth test, found that cats showed a higher frequency of exploratory and playful behaviors when accompanied by their owners, in comparison to when they were alone or accompanied by an unknown person [20, 21]. Similarly, those cats showed a lower frequency of alert and inactivity behaviors when their owners were present [20]. Another study verified an increase of affiliative behaviors in cats after reuniting with their owners [14]. All those studies revealed that cats express more security and stability in the presence of the owners, while in the owners’ absence they were more anxious and stressed. Therefore, it becomes relevant to study whether those animals can develop separation-related problems (SRP).

In the scientific literature, there is divergence regarding the nomenclature used for expressing the behavioral problems related to separation in companion animals with at least three terminologies commonly used: separation-related problems [22, 23]; separation distress [24] and separation anxiety syndrome [15, 25]. In spite of using different terms to describe this condition, some of the behaviors most commonly used to characterize SRP are usually the same: destructive behavior, excessive vocalization and inappropriate elimination when the animal is alone [22, 26]. In this study we will use the term SRP, since it is the most general and includes behavioral disturbances that occur in the presence or absence of physiological signs of stress [23, 24, 27].

Separation-related problems have been vastly studied in domestic dogs [23, 24, 27]; however, for cats few studies have reported the occurrence of SRP [15, 25, 28]. To the best of our knowledge, there are only two empirical studies [15, 28] and one review article [25] addressing this condition in cats. Studies that verify the care practices used by owners and the impacts of management on the welfare of cats are also scarce [6, 12, 29–31].

In the area of companion animal welfare, data provided from owners and/or caretakers are frequently used to estimate the prevalence rates of behavioral problems, behavioral signs of stress (like shaking, crying and excessive barks), use of aversive training methods and other conditions related to poor welfare [4, 32–34]. The majority of dog-focused SRP studies are based on questionnaire data [22, 35–37] since monitoring animals in domestic environments may not be viable.

Due to the importance of questionnaire studies, which enable the identification of relevant biological, social and cultural factors, this study aimed to develop a questionnaire for cat owners which identifies the most typical behaviors characteristic of SRP, as well as relates the occurrence of SRP to the management practices applied in the sampled cats. We hypothesized that *i*) the questionnaire will be able to identify behavioral signs reported by cat owners consistent with SRP; *ii*) animals that do not engage in intraspecific interactions and/or live in a restricted area and/or live in environments without enrichment will be more likely to be reported by owners as having behaviors consistent with SRP.

## Methods

### General view

This study was approved by the Juiz de Fora University Ethics Committee in Research with Human Beings, located in Juiz de Fora, Minas Gerais, Brazil, protocol # 2.084.228. The research participants signed a consent form before answering the questionnaire.

### Participants and recruitment

The interviewed population were owners of adult cats (above 6 months of age) residing in the city of Juiz de Fora, Minas Gerais State, Brazil. A total of 223 questionnaires were completed by 130 owners whose cats lived either in houses, apartments or commercial establishments. The snowball sampling method was used, in which the participants suggested new people to take part in the study. Recruitment of the initial sample of participants was achieved through use of social media, Facebook™, WhatsApp™ and Instagram™. Following recruitment, the researchers arranged meetings with the participants and completed the questionnaire during a semi-structured interview.

### Questionnaire

A questionnaire was developed based on published literature about separation anxiety syndrome in dogs [22, 23, 25, 26, 35, 36, 38–40] and cats [15, 25]. The initial part of the questionnaire was related to basic information about the animal as reported by the cat owners: name, breed, age, gender, reproductive status (neutered or not) and how long the owner had the cat.

The second part was related to the cat’s behavior when the owner was absent and/or visually separated from the cat. Therefore, questions related to the most typical behavioral signs of SRP were incorporated, including four behavioral categories (urination at inappropriate locations, defecation at inappropriate locations, destructive behavior and excessive vocalization) based on Schwartz [15]. Also, we defined three additional categories expressing mental states of the animals (depression, aggressiveness, agitation-anxiety) when the cat was alone or separated from the owner. The inclusion of these mental states was based on the assumptions that emotional health is a neglected subject especially in domestic cats [41] and that people can infer cats’ affective states by interpreting aspects of their facial expressions [41, 42]. The answers were ‘yes’ (Y) or ‘no’ (N) for each behavioral sign used.

Since previous studies have suggested that characteristics of the owner in addition to traits of the environment and management practices could affect the development of SRP in dogs and cats, the questionnaire included the following additional components: owner gender; owner age (in years); number of residents in the house (1, 2, 3, 4 to 7); number of female residents (none, 1, 2, 3 to 5); number of male residents (none, 1, 2, 3 to 5); type of residence (house or apartment); access to the whole house (Y, N); outdoor access (Y, N); frequency of outdoor access (always, often, occasionally, never); visual outdoor access (Y, N); access to elevated areas as in shelves, tables or others (Y, N); access to cat toys (Y, N); play with cat toys or other objects (Y, N, only when stimulated, does not have access to toys); frequency in which the cat was left alone in the house (5 to 7 times per week, 1 to 4 times per week, occasionally [i.e. less than once a week], never); duration for which the cat was left alone in the house (< 2 hours / day, 2 to 6 hours / day, > 6 hours / day, never left alone or do not know the answer); presence of other animals in the house (Y, N), change of behavior in the presence of an unfamiliar person (Y, N).

### Data analysis

A descriptive analysis of the questionnaire data was made through data categorization and calculating the frequency of each answer. After examining the frequencies of behaviors and emotional states indicative of SRP, cats were characterized as having possible SRP if they met the following criteria: I) cats for which the owners reported two or more behavioral categories used as indicators of SRP (urination at inappropriate locations, defecation at inappropriate locations, destructive behavior and excessive vocalization); II) cats with a positive answer for one behavioral category and one or more emotional states assessed; III) cats for which the owners reported the occurrence of three mental states indicative of SRP (depression, aggressiveness, agitation-anxiety). Cats assigned to one or more criteria defined by the authors were considered as the SRP group. Then, chi-square tests in contingency tables or Fisher’s exact tests for 2 x 2 tables were applied in order to verify associations between the demographic characteristics of cat population, owners’ characteristics and environmental or management traits with the occurrence of SRP. Data were processed using the software SAS (SAS Institute Inc., Cary, NC, version 9.2) with P < 0.05 for significance and P < 0.10 discussed as a tendency.

Dependences among variables were verified through Multiple Correspondence Analysis (MCA), which was used to reveal underlying patterns of associations between SRP and the answers regarding the owner characteristics, environmental and management traits. The MCA is an exploratory multivariate technique applied to strictly categorical variables useful for analyzing questionnaire data [43]. This multivariate technique allows exploration of the relationships between several categorical variables simultaneously, which can be expressed as “clouds” of points in a bidimensional space [43, 44]. MCA reveals the associations between each level of multiple categorical variables, allowing for determination of how the variables are related. This is the main advantage of MCA as compared to the chi-square test, which reveals significant associations between two variables only, and does not reveal the direction of association (i.e. how the variable categories are associated).

The MCA uses the chi-square in order to standardize frequencies and build the base for associations among the levels of the studied variables (named as correspondences) in a contingency table [45, 46]. It assigns scores on rows (corresponding to the subjects) and columns (corresponding to the answers’ categories) in a data matrix, creating charts [46]. All types of categorical variables are acceptable (nominal or ordinal, binary or with multiple levels) without distributional assumptions [43, 44]. Variance is expressed as the inertia, that is the dispersion of the data in relation to independence. The first dimension (Dim. 1) has the greatest proportion of the total inertia in the data set, followed by dimension 2 (Dim. 2), and so on. The distributions of the variables in both dimensions (Dim 1 vs. Dim 2) generates a biplot graph, where each variable category is represented by a point in the scatterplot. Closeness of points is interpreted as the association between rows and columns variables, revealing groups of correspondences [43, 44]. Thus, the MCA results were interpreted by the relative positions of the points and their distribution along the Dim. 1 and Dim. 2 axes. As categories become more related to SRP, the closer they were represented in space, falling in the same side or quadrant of the graphs. These analyses were performed using Statistica 7^®^ (7.0 version).

## Results

### Behavioral problems and occurrence of SRP

Among all sampled cats, 13.45% (30 / 223) met at least one of the three criteria we used to define SRP and they were owned by 25 different respondents (5 respondents had two cats meeting SRP criteria). Most of the SRP cats 90.00% (27 / 30) met criterion I (i.e. the owner reported two or more behaviors used as indicators of SRP); 70.00% (21 / 30) met criterion II (positive answer for one behavior and one or more emotional states); and 16.67% (5 / 30) met criteria III (the owners reported the three mental states indicative of SRP). Moreover, 50% (15 / 30) of cats met both criteria I and II; and 13.33% (4 / 30) of cats met all three criteria.

Regarding the behavioral / emotional signs in the total population studied (n = 223), depression during the owner’s absence was the most frequently reported sign, followed by excessive vocalization, agitation-anxiety and inappropriate elimination of urine (Table 1). The places where inappropriate elimination occurred were: owner’s bedroom floor and bed, below furniture in the living room, next to floor drains, carpets, sofas, plant vases, owner’s clothes and the kitchen sink. In the SRP group, the frequency of all behavioral signs indicative of SRP was higher than in the general population of cats (Table 1). Destructive behavior was the most reported sign in those cats, followed by urination in inappropriate places, excessive vocalization, agitation, depression-apathy, aggressiveness and, in lower frequency, defecation in inappropriate places.

**Table 1:** Absolute and relative frequencies (%, within parentheses) of behavioral / emotional signs of separation related problems (SRP) in the cat population sampled (total), in cats regarded as SRP, and in cats without indicators of SRP (Non-SRP).

Behavioral / emotional signs of SPR	Total	SRP	Non-SRP
	(n = 223)	(n = 30)	(n = 193)
Destructive behavior	33 (14.80)	20 (66.67)	13 (6.74)
Excessive vocalization	52 (23.32)	19 (63.33)	33 (17.10)
Elimination problems (urine)	23 (10.31)	18 (60.00)	5 (2.59)
Depression-apathy	58 (26.01)	16 (53.33)	42 (21.76)
Aggressiveness	22 (9.87)	11 (36.67)	11 (5.70)
Agitation-anxiety	39 (17.49)	11 (36.67)	28 (14.51)
Elimination problems (feces)	9 (4.04)	7 (23.33)	2 (1.04)

### Demographic characteristics of cat population and the occurrence of SRP

The age of the cats varied between 6 months to 16 years, with a mean of 3.9 ± 3.5 years. The cats’ characteristics (sex, age, neutering status and breed) were not related to SRP occurrence (P > 0.05, Table 2), except for time with the owner (χ^2^ = 9.23, P = 0.03).

**Table 2:** Absolute and relative frequencies (%, within parentheses) of the cat characteristics in the cat population sampled (total), in cats regarded as showing SRP, and in cats without indicators of SRP (Non-SRP). The results of chi-square test (or Fishers’ exact test in 2 x 2 tables) are shown to test the association between occurrences of SRP and the cats’ traits.

Cat characteristic	Total	SRP	Non-SRP	χ^2^	P-value
	(n = 223)	(n = 30)	(n = 193)		
**Sex**					
Male	89 (39.91)	14 (46.67)	75 (38.86)	-	0.43
Female	134 (60.09)	16 (53.33)	118 (61.14)		
**Age (years)**					
0.5 to 0.9	24 (10.76)	0	24 (12.44)	4.86	0.18
1.0 to 3.9	112 (50.22)	15 (50.00)	97 (50.26)		
4.0 to 7.9	60 (26.91)	10 (33.33)	50 (25.91)		
≥ 8.0	27 (12.11)	5 (16.67)	22 (11.40)		
**Time with the owner (years)**					
0.5 to 0.9	51 (22.87)	1 (3.33)	50 (25.91)	9.23	0.03
1.0 to 3.9	98 (43.95)	15 (50.00)	83 (43.01)		
4.0 to 7.9	50 (22.42)	11 (36.67)	39 (20.21)		
≥ 8.0	24 (10.76)	3 (10.00)	21 (10.88)		
**Had been sterilized**					
Yes	200 (89.69)	29 (96.67)	171 (88.60)	-	0.22
No	23 (10.31)	1 (3.33)	22 (11.40)		
**Breed**					
Purebred	27 (12.11)	3 (10.00)	24 (90.00)	-	0.78
Mixed breed	196 (87.89)	27 (12.44)	169 (87.56)		

### Association between owners’ characteristics and the occurrence of SRP

The number of residents varied from 1 to 7, with two or three people in most of the residences. Regarding the characteristics related to the residents, SRP occurrence was significantly associated with the number of females in the residence (χ^2^ = 12.37; P = 0.01). Most of the sampled residences had a single female (Table 3). Houses with two females had a higher occurrence of SRP than the rest of the sampled population (50.00% *vs*. 26.94% respectively). The owners who participated in the survey ranged in age from 18 to 75 years. The age and others owner characteristics (sex, number of residents and number of male residents) were not associated with the occurrence of SRP according to Fishers’ and chi-square tests (P > 0.05) (Table 3).

**Table 3:** Absolute and relative frequencies (%, within parentheses) of the owner characteristics in the cat population sampled (total), in cats regarded as showing SRP, and in cats without indicators of SRP (non-SRP). The results of chi-square test (or Fishers’ exact test in 2 x 2 table) are shown to test the association between occurrences of SRP and the owner characteristics.

Owner Characteristics	Total	SRP	Non-SRP	χ^2^	P-value
	(n = 223)	(n = 30)	(n = 193)		
**Sex**					
Male	39 (17.49)	5 (16.67)	34 (17.62)	-	1.00
Female	184 (82.51)	25 (83.33)	159 (82.38)		
**Age (years)**					
18 to 35	150 (67.26)	24 (80.00)	126 (65.28)	3.07	0.21
36 to 59	65 (29.15)	6 (20.00)	59 (30.57)		
≥ 60	8 (3.59)	0	8 (4.15)		
**Number of residents in the house**					
1	29 (13.00)	4 (13.33)	25 (12.95)	0.979	0.61
2 or 3	125 (56.05)	19 (63.33)	106 (54.92)		
4 to 7	69 (30.94)	7 (23.33)	62 (32.12)		
**Number of male residents**					
None	49 (21.97)	10 (33.33)	39 (21.21)	4.30	0.17
1	127 (56.95)	12 (40.00)	115 (59.59)		
2	47 (21.08)	8 (26.67)	39 (21.21)		
**Number of female residents**					
None	8 (3.59)	3 (10.00)	5 (2.59)	12.37	0.01
1	108 (48.43)	8 (26.67)	100 (51.81)		
2	67 (30.04)	15 (50.00)	52 (26.94)		
3 to 5	36 (17.94)	4 (13.33)	36 (18.65)		

**Fig 1:**
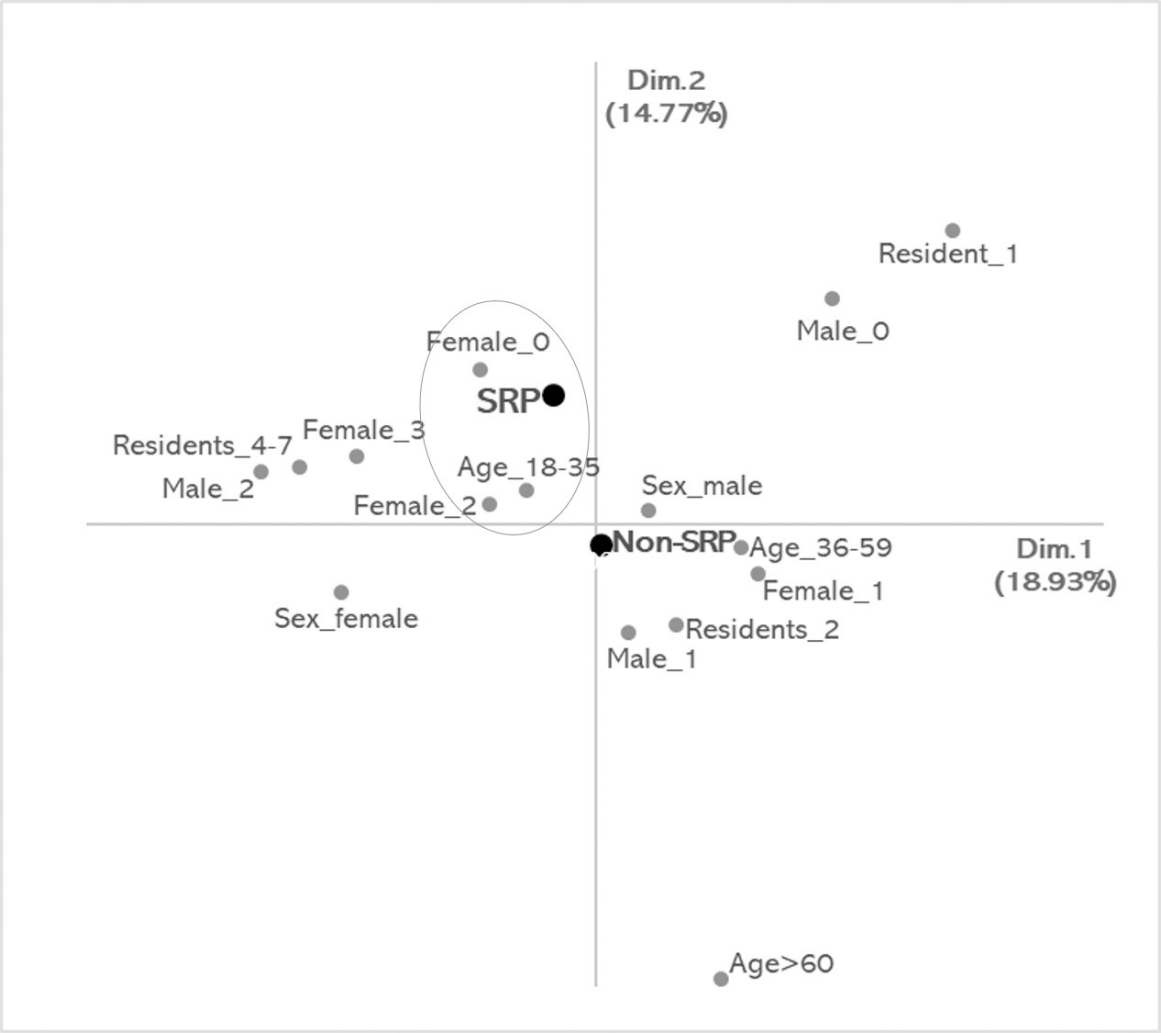
Perceptual map of the multiple correspondence analyses for separation related problems (SRP) and the owner characteristics. Grey circle represents the correspondences among the variable categories SRP, ‘no female residents’ (Female_0), ‘two female residents’ (Female_2) and ‘age 18 to 35 years' (Age_18–35).

The MCA generated two dimensions, the first (Dim. 1) accounted for 18.93% of the inertia (eigenvalue: 0.35) and the second (Dim. 2) for 14.77% (eigenvalue: 0.27), yielding a cumulative variance of 33.70%. In Dim. 1 the variable with highest positive contribution to inertia was ‘one resident’ and the variables with highest negative contributions were ‘two male residents’, and ‘4 to 7 residents’ (Fig 1). In Dim. 2, ‘one resident’ and ‘no male resident’ had positive contributions and ‘age ≥ 60’ had the highest negative contribution (Fig 1). Based on the visual analysis of the MCA perceptual map, it was possible to identify that the ‘non-SRP’ category was positioned near the origin (center of the graph). Thus, it did not reveal interpretable patterns of association with the owner traits that deviate from independence. In turn, the ‘SRP’ category was located in quadrant IV of the graph, and revealed an interpretable group of correspondence (associations) of SRP with ‘no female resident’, ‘two female residents’ and ‘age 18 to 35 years' owner characteristics (Fig 1). Based on the closeness among the points of this group, cats whose owners reported behaviors consistent with SRP were associated with households including no female residents, owners aged 18 to 35 years, and two female residents.

### Association between environmental or management traits and the occurrence of SRP

Among the environmental traits assessed, playing with toys showed a significant association with occurrence of SRP (χ^2^ = 8.30; P = 0.04), in which SRP occurred more in cats that had no access to toys compared to the total population sampled (Table 4). The SRP occurrence was also associated with the presence of other animals in the house (Fisher’s exact test, P = 0.04). Residences with no other animals had a higher percentage of cats with SRP signs than the non-SRP group (30.00% *vs*. 14.51% respectively) (Table 4).

**Table 4:** Absolute and relative frequencies (%, within parentheses) of the environmental and management traits for the cat population sampled (total), in cats regarded as SRP, and without signs of SRP (Non-SRP). The results of chi-square test (or Fishers’ exact test in 2 x 2 table) are shown to test the association between occurrences of SRP and environmental or management traits.

Environment or management	Total	With SRP	Non-SRP	χ^2^	P-value
	(n = 223)	(n = 30)	(n = 193)		
**Type of residence**					
House	126 (57.01)	14 (46.67)	112 (58.64)	-	0.24
Apartment	95 (42.99)	16 (53.33)	79 (41.36)		
**Access to the whole house**					
Yes	175 (78.48)	21 (70.00)	154 (79.79)	-	0.24
No (cat is restricted in a single room)	48 (21.52)	9 (30.00)	39 (20.21)		
**Outdoor access**					
Yes	177 (79.37)	21 (70.00)	156 (80.83)	-	0.22
No (kept exclusively indoors)	46 (20.63)	9 (30.00)	37 (19.17)		
**Frequency of outdoor access**					
Always	39 (17.49)	4 (13.33)	35 (18.13)	1.88	0.597
Oftenly	7 (3.14)	2 (6.67)	5 (2.59)		
Occasionally	27 (12.11)	3 (10.00)	24 (12.44)		
Never	150 (67.26)	21 (70.00)	129 (66.84)		
**Visual outdoor access**					
Yes	187 (83.86)	23 (76.67)	164 (84.97)	-	0.28
No	36 (16.14)	7 (23.33)	29 (15.03)		
**Access to elevated areas**					
Yes (in shelves, tables or others)	185 (82.96)	26 (86.67)	159 (82.38)	-	0.62
No	38 (17.04)	4 (13.33)	34 (17.62)		
**Access to cat toys**					
Yes	185 (82.96)	22 (73.33)	163 (84.46)	-	0.19
No	38 (17.04)	8 (26.67)	30 (15.54)		
**Play with toys (cat toys or objects)**					
Yes	113 (50.67)	13 (43.33)	100 (51.81)	8.30	0.04
No	28 (12.56)	1 (3.33)	27 (13.99)		
Only when stimulated	54 (24.22)	8 (26.67)	46 (23.83)		
No access to toys	28 (12.56)	8 (26.67)	20 (10.36)		
**Left alone in the house (frequency)**					
5 to 7 times per week	109 (48.88)	18 (60.00)	91 (47.15)	6.51	0.09
1 to 4 times per week	40 (17.94)	8 (26.67)	32 (16.58)		
Occasionally (less than once a week)	50 (22.42)	3 (10.00)	47 (24.35)		
Never	24 (10.76)	1 (3.33)	23 (11.92)		
**Left alone in the house (duration)**					
< 2 hours / day	25 (11.21)	2 (6.67)	23 (11.92)	5.58	0.13
2 to 6 hours / day	83 (37.22)	16 (53.33)	67 (34.72)		
> 6 hours / day	86 (38.57)	11 (36.67)	75 (38.86)		
Not left alone or do not know	29 (13.00)	1 (3.33)	28 (14.51)		
**Other animals in the house**					
Yes	186 (83.41)	21 (70.00)	165 (85.49)	-	0.04
No	37 (16.59)	9 (30.00)	28 (14.51)		
**Change with unfamiliar person**					
Yes	121 (54.26)	16 (53.33)	105 (54.40)	-	1.00
No	102 (45.74)	14 (46.67)	88 (45.60)		

**Fig 2:**
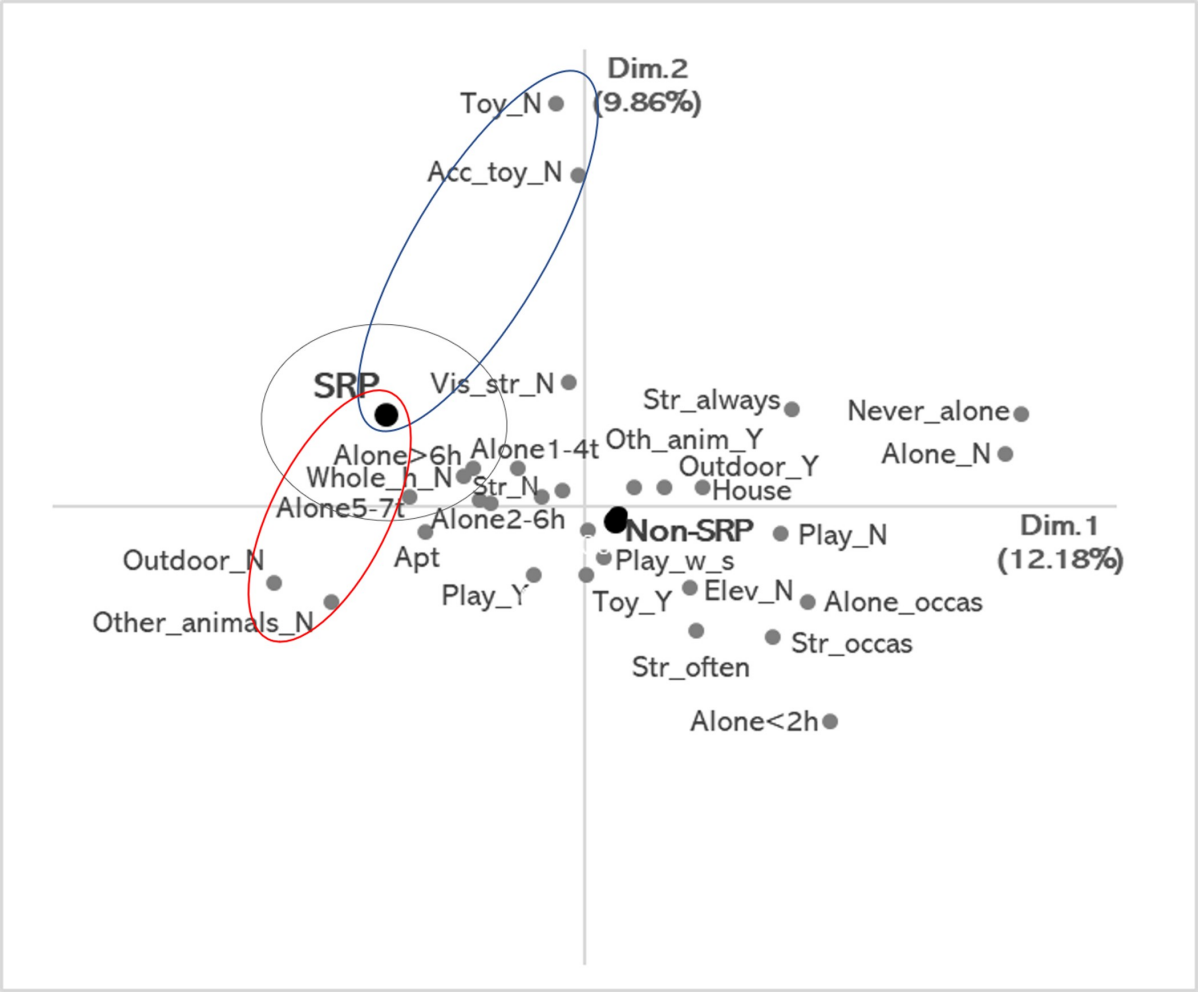
Perceptual map of the multiple correspondence analyses for separation related problems (SRP) and the owner characteristics. Grey circle represents the correspondences among the variable categories ‘SRP’, ‘left alone in the house > 6 hours / day’ (Alone>6h), ‘no access to the whole house’ (Whole_h_N) and ‘left alone in the house 5 to 7 times per week’ (Alone5-7t). Red circle represents the correspondences among ‘SRP’, ‘no other animals in the house’ (Other_animals_N) and ‘no outdoor access’ (Outdoor_N). Blue circle represents the correspondences among ‘SRP’, ‘no access to cat toys’ (Acc_toy_N) and ‘no access to toys’ (Toy_N).

Two dimensions were retained in the MCA, Dim. 1 accounted for 12.18% of the inertia (eigenvalue: 0.20) and Dim. 2 accounted for 9.86% (eigenvalue: 0.16), yielding a cumulative variance of 22.04%. In Dim. 1 the variable with highest positive contribution to inertia was ‘never left alone in the house (frequency)’ and ‘not left alone or do not know (duration)’, with the highest negative contribution for ‘no outdoor access’ (Fig 2). In Dim. 2, ‘no access to cat toys’ and ‘no access to toys’ had the highest positive contributions and ‘left alone in the house < 2 hours / day’ had the highest negative contribution (Fig 2). Based on the visual analysis of the MCA perceptual map, it was possible to identify that the ‘non-SRP’ category was positioned near the center of the graph and, thus, did not reveal associations that deviate from independence. The SRP category was positioned in the IV quadrant and showed three interpretable correspondence groups. Based on the closeness among the points in the IV quadrant, the first interpretable group of correspondences was composed by ‘SRP’, ‘left alone in the house > 6 hours / day’, ‘no access to the whole house’ and ‘left alone in the house 5 to 7 times per week’. In Dim 1 a second group of correspondences was ‘SRP’, ‘no other animals in the house’ and ‘no outdoor access’. In Dim 2. a third interpretable correspondence group was ‘SRP’, ‘no access to cat toys’ and ‘no access to toys’ (Fig 2).

## Discussion

Most studies about cat behavior have been done under experimental conditions (laboratories), in shelters, or in feral cat colonies; thus, there is a gap in the knowledge regarding the behavior of domiciled cats and the interactions with their owners [47–50]. This study provides information about behavioral signs consistent with SRP in a sampled population of domestic cats, as well as about the management practices used by their owners. The questionnaire identified that about 13% of cats may have signs consistent with SRP according to their owners’ reports, and therefore, it could be a promising tool for future research into investigating SRP in cats. We also found elements related to the owner as well as environmental and management characteristics that may predispose cats to be reported by owners as having signs consistent with SRP.

Cats might be regarded as social partners for their owners and vice-versa [51]. For instance, a previous study found temporal patterns of interaction between owners and their cats. Those patterns vary depending on factors that influence the human-cat bond and relationship, such as the owners and cats personalities and owners sex [51]. For example, the more extroverted the owner’s personality, the higher the frequency of interactions with their cats. Moreover, in dyads with a female owner, the number of interactions per minute was higher when compared to dyads with a male owner [51]. In general, both domiciled and shelter cats can benefit from human contact and they seek it through affiliative behaviors [19, 47, 51, 52]. Therefore, it is essential to investigate the possibility of SRP occurrence in domestic cats, given that some studies suggest that cats develop attachment and secure bonding with their owners [20, 51, 53]. For instance, a study found indicators of attachment relationships between humans with their kittens and adult cats, including proximity seeking, separation distress and reunion behavior, as well as individual differences were consistent with attachment style categorizations [53].

In the present study, 30 of 223 evaluated cats (13.45%) were classified as possibly SRP-affected based on the behavioral signs reported by their owners to occur during their absence. A previous empirical study found a prevalence of 19% (n = 136) of cats affected by SRP in a group of 716 animals [15], considering as SRP cats showing one or multiple behavioral signs displayed exclusively in the absence of the attachment figure: inappropriate urination (96 cats), inappropriate defecation (48), excessive vocalization (16), destructiveness (12), and psychogenic grooming (8 cats). Together, these results reveal the likelihood of SRP occurrence in cats along with a gap of information regarding SRP in the species, suggesting this is a neglected issue in the area of behavioral problems in cats. The Fe-BARQ online questionnaire, developed to measure owner-reported behavior in domestic cats, is an extensive list with 149 behavioral questions/items encompassing multiple behavioral factors, most of which capture behavioral problems [4]. Separation-related behaviors are evaluated by six items, including behaviors of ‘restlessness–agitation’, ‘hide and/or slink away’, ‘lie down or stay still’, ‘active investigation’, ‘alert/hyper-vigilance’ and ‘vocalization’ just prior to or during cat separation from the owner [4]. For assessment of SRP, the Fe-BARQ did not include the most typical signs of destructive behavior and inappropriate elimination of urine and feces both exclusively occurring in the absence of the owner, as did the present study. Vocalization when the cat was left alone was included in both questionnaires. While the Fe-BARQ was based on factor analysis from a large sample of respondents (n = 2608), the incidence of each behavioral item indicative of SRP was not reported, nor was the prevalence of possible SRP in the sample [4]. Given the lack of information on cats, the literature on SRP in dogs can be useful for general comparisons. The cat SRP prevalence in the present study was within the range reported in previous studies assessing SRP in dogs: 13% in Dinwoodie et al. [54]; 17.2% in Tiira et al. [40]; 20% in Martínez et al. [55]; 22.58% in Storengen et al. [38]; 18.4% to 33.1% in Konok et al. [56]; 30% in Blackwell et al. [57]. In most of these studies, the identification of SRP was based on the reports of dog owners (interviews and questionnaires).

It is worth noting that the SRP cats of the present study were reported by their owners as having behavioral or emotional signs consistent with SRP (defined here as SRP group) and did not necessarily have SRP, as the questionnaire still needs further validation based on behavioral observations or experimentation. In addition, none of the owners reported that their cats had any previous diagnosis of SRP by a veterinarian or clinical ethologist. The behaviors and mental states reported in the present study may also indicate other disorders such as generalized anxiety, boredom, or physiological problems. In fact, in a study about separation anxiety in dogs [23], several behaviors observed (inadequate elimination, excessive vocalization and self-mutilation behaviors) were nonspecific and also seen in the control group (dogs without separation anxiety). However, in animals without separation anxiety these behaviors occurred in both the presence and in the absence of the owner [23]. Despite not being able to rule out that the interviewed owners answered the questionnaire based on more general behaviors, during the interviews owners were informed that the signs had to be displayed during owners’ absences. One potential problem with this is the possibility of owners not having a valid perception of the behavior and mental states of their cats when they were not present to observe them. However, we should infer that the owners answered based on evidence such as the behavior or body language of the cat when they were absent, which could be based on reports by others residents, neighbors or any kind of sign the cat left in the environment (feces, urine or broken objects). This methodological limitation is difficult to overcome in a questionnaire survey, since the unequivocal view of the cat body language during owners’ absence could only be obtained by regular video monitoring of the cats when left alone, which has a low feasibility. A study conducted with dogs addressed the shortcomings of the methodologies based on owners reports to assess separation behaviors [58], whereby a separation-related behavioral score based on owners reports was correlated with dogs behaviors based on video footage of the dogs during the first 25 minutes after they were left alone in the house [58]. Thus, it is reasonable to infer that the respondents of our study had different ways to gather evidence about their cats’ behaviors during the owners’ absence.

To consider a cat as possibly having SRP, the owner had to report at least two behaviors characteristic of this condition: destructive behavior, inappropriate elimination of urine, inappropriate elimination of feces, excessive vocalization, necessarily occurring during the absence of the owner. In the group of animals characterized as SRP group, destructive behavior was the most prevalent sign, demonstrated by 66.67% of the cats, as opposed to a 6.74% prevalence in the group of animals with no behavioral signs of SRP (non-SRP). This is one of the most frequently reported behaviors as a symptom of SRP for both cats [15] and dogs [23, 25]. In a study evaluating 200 dogs with SRP and its possible risk factors, destructive behavior was demonstrated by 71.7% of the total sample [23]. Nevertheless, in the present study it is not possible to rule out that the high frequency of destructive behavior reported by the cat owners occurred due to a mistaken perception about scratching behavior. Some of the interviewed owners may not have differentiated natural scratching behavior from abnormal destructive behavior (i.e., when it is shown to a frequent and exaggerated extent). Behavioral problems might be perceived as any behavior shown by the animal that is unacceptable for the owner, but some of them may be natural, such as scratching [9, 59].

Excessive vocalization is a common sign in dogs with separation anxiety [23]. As previously mentioned, for adult cats vocalization is an indicator of stress [60] and also of SRP [4, 15] and, as such, it was included in the questionnaire. We obtained a prevalence of 63.33% for this behavior in the sample, making this the second most reported sign for owners of cats from SRP group. Excessive vocalization can be considered an easily perceived behavior, since it may cause disturbance to other residents and the neighborhood. Despite being easily perceived, it is a non-specific behavioral symptom and potentially indicative of other problems, e.g. cognitive dysfunction syndrome [61].

Regarding inappropriate urination, 60% of the cats defined here as belonging to the SRP group showed this behavior. This is one of the most characteristic signs of SRP, showing high prevalence in previous studies [15, 62]. In the single study we found about separation anxiety in cats, Schwartz [15] found a prevalence of 70.6% for inappropriate urination in a sample of 136 cats with separation anxiety. It has been suggested that inappropriate urination in the absence of the owner could be the only behavioral sign of SRP for cats [15], even when not combined with other evident behaviors and physiological symptoms [62]. It may be usual for urine to be eliminated in places where there is presence of the owner’s smell, such as bed, clothes, pillows and shoes [62]. However, it is not possible to guarantee that cat owners are able to distinguish inappropriate urination as a sign of SRP from normal territorial marking with urine. Territory marking by urine (or spray) is a normal feline behavior which tends to happen on vertical surfaces, independent of the presence of the owner in the home. To avoid this misunderstanding, in the present study during the questionnaire it was reinforced that inappropriate elimination was only considered when it occurred in the owners’ absence.

There was a higher frequency of elimination of urine in inappropriate places than inappropriate defecation (60.0% *vs*. 23.3%, respectively) in cats characterized as possibly having SRP. In a previous study, the frequency of inappropriate defecation was higher, occurring in 35.3% of 136 cats with SRP [15]. Such differences in frequencies of inappropriate defecation may have occurred because the study by Schwartz [15] was based on medical records, so we could infer that inadequate defecation could be a symptom that motivated the owners to seek medical assistance and/or could also be related to some underlying disease. Defecation is also a non-specific indicator of SRP that can occur in conjunction with other behavioral problems or pathological causes [63].

In addition to the behavioral categories previously described as SRP indicators, we included three questions related to owner-perceived cat emotional states, including depression-apathy, agitation-anxiety and aggressiveness. Among those signs the most prevalent was depression-apathy, which occurred in approximately half of the cats belonging to the SRP group. The higher frequency of depression-apathy could indicate that this was a more adequate subjective sign of SRP compared to the other states included in the questionnaire. However, it is also plausible that cat owners had a misperception of their cats’ body language, since they are animals with nocturnal habits and long periods of sleep and inactivity during the day [59, 64], which coincides with the period that owners leave home for work. For example, there is evidence that dog owners are able to perceive more evident signs of stress, such as trembling, whining, aggressiveness, excessive barking, and panting, but are rarely able to perceive signs of stress characterized as ‘subtle behaviors’ such as looking elsewhere, turning head, yawning, and nose licking [65]. Additionally, in a study aimed at identification of cats’ facial expressions by humans, it was found that some people can correctly infer the affective states of cats from subtle aspects of their facial expressions [42]. Thus, the lower prevalence of the other two behavioral signs included in the present study could be related to those being less perceptible or more tolerable to cat owners, and thus unnoticed by them. More research is needed to determine to what extent owners are able to perceive emotional states and subtle signs of stress and anxiety from their cats.

In the scientific literature characteristics like gender, age, and neutering status have been reported as risk factors for SRP in dogs [27]. As well, Separation Anxiety Syndrome was more commonly reported in senior female cats than in males, with a prevalence of 27% in females aged 7 years or more [15]. Additionally, destructive behavior was reported as more frequent in neutered male cats whereas inappropriate defecation was more prevalent in neutered females [15]. However, in this study we found no relationship between cat sex and neuter status with any symptoms consistent with SRP reported by the owners. Most of the cats assessed in the present study were sterilized (89.69%), with only 23 intact individuals included.

Some previous studies also suggested that breed can be related to SRP in dogs [23, 26, 38]. The process of artificial selection for some breeds could explain, in part, the higher susceptibility of SRP in certain breeds [66]. A recent study showed that dog breeds selected for cooperative work with humans were more prone to suffer from separation-related stress behaviors than the breeds selected for independent work abilities [66]. For cats, it was previously suggested that Siamese and Burmese breeds are more prone to developing SRP [15, 62]. There is empirical evidence that Siamese, Burmese and Tonkinese coat patterns are also related to the occurrence of SRP and separation anxiety [28]. The shortage of studies assessing breed effects on the risk of SRP in companion animals may be due to methodological restraints for developing reliable assessments of this question, given the requirement for a large number of animals of different breeds to estimate the SRP prevalence in various dog [40] and cat breeds. For instance, in the present study only 12.11% of the cats were purebred. However, we did not evaluate whether cats reported as purebreds by their owners were indeed purebred based on pedigree information, what could lead to even lower percentages.

As for the owners’ traits, a positive association was observed between the report of signs of SRP and the presence of ‘no female resident’ according to MCA and ‘two female residents’ in the house. In cats included in the SRP group, 10.00% of them lived in residences with ‘no female’, while in non-SRP the frequency was much lower at 2.59%. In addition, for the SRP group, 50.00% of cats lived with two females, while in the non-SRP group 26.94% did so. Thus, the relationships found between SRP and number of female residents in the present study were not straightforward and are difficult to explain. Previous findings in dogs had already reported a relationship between SRP and the number of female residents: as the number of females in the house increased, so did the likelihood of the dog developing SRP [35]. Additionally, dogs owned by a single woman were more prone to SRP than those raised by a single man [38]. The reason for this difference is not clear; however, there is some evidence from previous studies suggesting that female owners show more attachment to their pets than male owners, and cats prefer to interact with adult female residents than with children and male adults [33, 51, 67]. As an alternative explanation, it is also plausible that women showed higher perception of their pets’ behavior and body language, and thus owner sex is not necessarily a factor that makes the animal more prone to SRP, but potentially makes the owner more perceptive of SRP signs [33, 67]. Still, regarding the owners’ traits assessed in the present study, the MCA correspondence grouping related to SRP also included the variable ‘owners’ age 18 to 35’. We might infer that this age can be confounded with other traits such as number of female residents and time that cats are left alone for younger owners.

Regarding the environmental and management traits, the more cats’ popularity as a pet grows, the greater the need for better management practices and responsible ownership [68]. Responsible cat ownership includes practices that protect those animals from damage and behavioral problems, increasing their welfare. In the present study, cats reported by owners as having behaviors consistent with SRP were related to ‘do not have access to toys’, ‘do not have access to the whole house’, ‘no other animal in the house’, ‘no outdoor access’ and being left alone in the house ‘5 to 7 times per week’, ‘from 2 to 6 hours per day’ and ‘> 6 hours per day’. The confined environments typical of residences usually do not meet the exploratory needs of cats, because they may not provide the stimuli the animal would find in the wild, which makes the environment monotonous and predicable [69]. Thus, environmental enrichment benefits confined cats, helping to reduce the stress caused by confinement, any abnormal behaviors, encouraging exploratory behavior and other uses of the space [69]. In the total population of cats, 12.56% of the animals had no access to toys while in the SRP group 26.67% of them had no access to toys, making this a possible factor related to SRP. Therefore, the use of environmental enrichment, such as cat toys, can be a good option to increase the welfare of confined animals and help to prevent SRP [25, 26, 69, 70].

It was also observed that the frequency and duration of daily periods the animal is separated from its attachment person can be related to the report of signs of SRP by the owners, especially for cats that stay alone from 5 to 7 times a week and more than 2 hours per day, as revealed by MCA. Additionally, the frequency that the cats are left alone tended to be related with reported signs of SRP, according to the chi-square test. In the sample of cats without SRP, there was a lower percentage (47.15%) of animals that stayed alone from 5 to 7 times a week than in the SRP group (60.0%). In the SRP group, only 3.33% of the cats never stayed alone or rarely stayed alone (10.0%), while those percentages were much higher in the total population sampled (10.76% and 22.42% respectively), indicating that cats not left alone are less likely to develop SRP. Cats are considered animals that can easily tolerate the absence of their owners [14]. We speculate that owners who perceive their cats as ‘independent’ animals might leave them alone for longer periods of time, contributing to the occurrence of SRP in cats that stay alone for long periods. Further studies should investigate the relationships among owners’ perceptions towards cat behavior and the management practices applied that predispose to SRP.

We also observed a tendency for owners reporting signs of SRP in the group of individuals that do not live with other animals at home. A possible explanation for this association is that cats living alone spend more time interacting with their owners than those living with other cats. It is also possible that owners with a single cat ‘spoiled’ their animal more than those with multiple cats [4, 71]. However, these two possibilities lack scientific support and warrant more research. The simple presence of other cats in the house may not be considered a factor that would prevent the occurrence of SRP in cats [14]. While some authors suggest that multi-cat households can be stressful [72], others indicate that there is no significant difference in stress scores between cats from single-cat and those from multi-cat households [73]. In some cases, having another animal in the environment may be beneficial for certain cats depending on their temperament, since they could maintain positive interactions without agonistic confrontations [64].

In spite of promising results, this study has limitations that must be acknowledged. The interviewed owners were asked about behavioral signs of SRP in their absence (i.e., in absence of the presumed attachment figure), without recording whether the behaviors also occurred when someone else was in the house or exclusively when the animal was alone. This information could elucidate whether those behaviors were more related to general isolation than to the absence of the owner per se. An additional point is that the three criteria used to characterize SRP were arbitrarily defined by the authors. Using a combination of behaviors and mental states to define SRP, we have found a slightly lower incidence than a previous study (13% *vs*. 19% in Schwartz [15]) in which a single behavioral sign of SRP displayed when cats were separated from the owner were enough to cats being regarded as affected [15]. The prevalence of SRP in a sampled population can be dependent on the criteria used to diagnose SRP, generating a potential source of bias, subjectivity or imprecision that has to be taken into account in future studies. To date, very few exploratory researches were conducted about SRP in cats. The lack of science-based criteria to define SRP in cats reinforce that separation problems can be regarded as a neglected feline behavioral problem that deserves more studies.

## Conclusion

Separation-related problems in domestic cats are behavioral disorders that are difficult to identify due to the very limited amount of research conducted to date. The present questionnaire enabled the identification of behaviors (destructive behavior, excessive vocalization, inappropriate elimination of urine) and mental state (depression-apathy) related to SRP in cats, as reported by their owners. Even though the questionnaire cannot be used as a substitute for a detailed investigation of each case, it can be used as a starting point for future research about SRP in cats. It may provide a practical and efficient instrument to help ethologists and veterinarians make initial diagnoses of SRP with more confidence.

Through this study we suggest that some environmental factors can make domestic cats more prone to develop separation-related problems, like the number of female humans in the house, frequency and number of daily hours the cat is left alone, the lack of use of environmental enrichment (e.g. toys), and the absence of other animals in the house. Thus, investigations of management practices to prevent the occurrence SRP should take these factors into consideration.

## Supporting information

S1 File Questionnaire for cat owners. (DOCX)

S1 Table Dataset used in statistical analyses (n = 223). (XLSX)

S2 Table Results of the multiple correspondence analyses (MCA) for separation related problems (with SRP) or without SRP (non-SRP) and owners characteristics (n = 223). Values of coordinates, inertia and Cosine2 in dimension 1 (Dim. 1) and dimension 2 (Dim. 2) are shown.
(DOCX)

S3 Table Results of the multiple correspondence analyses (MCA) for separation related problems (with SRP) or without SRP (non-SRP) and environmental and management characteristics (n = 223). Values of coordinates, inertia and Cosine2 in dimension 1 (Dim. 1) and dimension 2 (Dim. 2) are shown.
(DOCX)
