# Supplementary material for: Identification of separation-related problems in domestic cats: A questionnaire survey
Source: PLoS One. 2020 Apr 15;15(4):e0230999. doi: 10.1371/journal.pone.0230999 (PMC7159185; doi:10.1371/journal.pone.0230999)
Supplement: S1 File — (DOCX) [file pone.0230999.s001.docx]

**Identification of behavioral signals of separation related problems in domestic cats**

**Questionnaire for cat owners**

**Owner’s information:**

1. Name: ___________________________________________________________________________
2. Owner gender: ( ) male / ( ) female.
3. Age (years): ______________________________________________________________________
4. How many people living in the house: ( ) male / ( ) female / ( ) children / ( ) total.

**Cats’ information:**

1. Name of the cat: ___________________________________________________________________
2. Cat sex: ( ) male / ( ) female.
3. Age (months or years): ______________________________________________________________
4. For how long have you been its owner? _________________________________________________
5. Is it neutered? ( ) yes / no ( ).
6. Is the cat mixed breed ( ) or purebred ( )? If purebred, what is the breed_____________________

**Answer about cat behavior when the owner (i.e. attachment figure) is absent. Just answer YES if the behavioral signal occurs when you are not in the house or visually separated from the cat (it perceives you as absent):**

1. Does the cat vocalize constantly, with cries and sharp meows, in the absence of the attachment figure? ( ) yes / ( ) no.
2. Does the cat destroy the furniture or other objects in the absence of the attachment figure?

( ) yes / ( ) no.

1. Does the cat urinate in inappropriate places in the absence of the attachment figure?

( ) yes / ( ) no.

a) If yes, where does it urinate? _______________________________________________________

1. Does the cat defecate in inappropriate places in the absence of the attachment figure?

( ) yes / ( ) no

a) If yes, where does it defecate? ______________________________________________________

1. Does the cat show itself agitated and moving too much in the absence of the attachment figure, with signals of agitation-anxiety? ( ) yes / ( ) no.
2. Does the cat show sadness or depression in the absence of the attachment figure, that is, with signals of depression-apathy? ( ) yes / ( ) no.
3. Does the cat show aggressiveness in the absence of the attachment figure? ( ) yes ( ) no.

**Cat environment and management**

1. Where does your cat live in? ( ) house / ( ) apartment / ( ) commercial establishment.
2. Does the cat have access to all rooms of the house during the owners’ absence? ( ) yes / ( ) no.
3. Does the cat have outdoor access, for example, a garden, backyard or balcony? ( ) yes / ( ) no (the cat is kept exclusively indoor).
4. How often does it have access to the street? ( ) always / ( ) oftenly / ( ) occasionally /

( ) never.

1. Does your cat have visual access to street through glasses or windows? ( ) yes / ( ) no
2. Does your cat have access to elevated areas such as shelves or other high places in the house?

( ) yes / ( ) no.

1. Does your cat have any toys or objects that it uses as toys? ( ) yes ( ) no ( ). If yes, to what type of objects?_____________________________________________________________________
2. Does it like to play with cat toys (as environmental enrichment items)? ( ) yes ( ) no ( ) only when it is stimulated.
3. How often is he cat left alone in the house? ( ) 5 to 7 times per week / ( ) 1 to 4 times per week / ( ) occasionally (less than once a week) / ( ) never.
4. How long is the cat left alone in the house? ( ) less than 2 hours per day / ( ) from 2 to 6 hours per day / ( ) more than 6 hours per day / ( ) not left alone or do not know.
5. Are there other animals in the residence? ( ) yes / ( ) no. If yes, how many? ______________________________________________________________________________
6. If the previous answer was “yes”, does your cat have contact with the other animals? ( ) yes / ( ) no.
7. Does your cat change its behavior in the presence of unfamiliar people, for example when you receive visits? ( ) yes / ( ) no.
