# Supplementary material for: Identification of separation-related problems in domestic cats: A questionnaire survey
Source: PLoS One. 2020 Apr 15;15(4):e0230999. doi: 10.1371/journal.pone.0230999 (PMC7159185; doi:10.1371/journal.pone.0230999)
Supplement: S2 Table — Values of coordinates, inertia and Cosine2 in dimension 1 (Dim. 1) and dimension 2 (Dim. 2) are shown. (DOCX) [file pone.0230999.s003.docx]

**S 3.** Results of the Multiple Correspondence Analyses (MCA) for separation related problems (with SRP) or without SRP (non-SRP) and owners characteristics. Values of coordinates, inertia and Cosine^2^ in dimension 1 (Dim. 1) and dimension 2 (Dim. 2) are shown.

| **Owner characteristic** | **Coordin.**  **Dim. 1** | **Coordin.**  **Dim. 2** | **Inertia**  **Dim. 1** | **Inertia**  **Dim. 2** | **Cosine²**  **Dim. 1** | **Cosine²**  **Dim. 2** |
| --- | --- | --- | --- | --- | --- | --- |
| **With SRP** | -0.164 | 0.731 | 0.002 | 0.044 | 0.004 | 0.083 |
| **Without SRP** | 0.026 | -0.114 | 0.000 | 0.007 | 0.004 | 0.083 |
| **Sex** |  |  |  |  |  |  |
| Male | -0.998 | -0.379 | 0.084 | 0.015 | 0.211 | 0.030 |
| Female | 0.211 | 0.080 | 0.018 | 0.003 | 0.211 | 0.030 |
| **Age (years)** |  |  |  |  |  |  |
| 18 to 35 | -0.274 | 0.191 | 0.024 | 0.015 | 0.154 | 0.075 |
| 36 to 59 | 0.572 | -0.127 | 0.046 | 0.003 | 0.134 | 0.007 |
| ≥ 60 | 0.496 | -2.554 | 0.004 | 0.144 | 0.009 | 0.243 |
| **Number of residents** |  |  |  |  |  |  |
| 1 | 1.408 | 1.658 | 0.124 | 0.220 | 0.296 | 0.411 |
| 2 or 3 | 0.315 | -0.563 | 0.027 | 0.109 | 0.127 | 0.405 |
| 4 to 7 | -1.163 | 0.324 | 0.201 | 0.020 | 0.606 | 0.047 |
| **Number of male resident** |  |  |  |  |  |  |
| None | 0.929 | 1.276 | 0.091 | 0.220 | 0.243 | 0.459 |
| 1 | 0.129 | -0.604 | 0.005 | 0.128 | 0.022 | 0.482 |
| 2 | -1.316 | 0.300 | 0.175 | 0.012 | 0.462 | 0.024 |
| **Number of female resident** |  |  |  |  |  |  |
| None | -0.453 | 0.871 | 0.004 | 0.017 | 0.008 | 0.028 |
| 1 | 0.641 | -0.278 | 0.096 | 0.023 | 0.386 | 0.073 |
| 2 | -0.419 | 0.113 | 0.025 | 0.002 | 0.075 | 0.005 |
| 3 to 5 | -0.939 | 0.388 | 0.076 | 0.017 | 0.193 | 0.033 |
