# Supplementary material for: Identification of separation-related problems in domestic cats: A questionnaire survey
Source: PLoS One. 2020 Apr 15;15(4):e0230999. doi: 10.1371/journal.pone.0230999 (PMC7159185; doi:10.1371/journal.pone.0230999)
Supplement: S3 Table — Values of coordinates, inertia and Cosine2 in dimension 1 (Dim. 1) and dimension 2 (Dim. 2) are shown. (DOCX) [file pone.0230999.s004.docx]

**S 4.** Results of the Multiple Correspondence Analyses (MCA) for separation related problems (with SRP) or without SRP (non-SRP) and environmental and management characteristics. Values of coordinates, inertia and Cosine^2^ in dimension 1 (Dim. 1) and dimension 2 (Dim. 2) are shown.

| **Environment or management** | **Coordin. Dim. 1** | **Coordin. Dim. 2** | **Inertia**  **Dim. 1** | **Inertia**  **Dim. 2** | **Cosine²**  **Dim. 1** | **Cosine²**  **Dim. 2** |
| --- | --- | --- | --- | --- | --- | --- |
| **With SRP** | -0.745 | 0.504 | 0.029 | 0.016 | 0.086 | 0.039 |
| **Non-SRP** | 0.116 | -0.078 | 0.005 | 0.003 | 0.086 | 0.039 |
| **Type of residence** |  |  |  |  |  |  |
| Home | 0.445 | 0.101 | 0.045 | 0.003 | 0.267 | 0.014 |
| Apartment | -0.600 | -0.137 | 0.060 | 0.004 | 0.267 | 0.014 |
| **Access to the whole house** |  |  |  |  |  |  |
| Yes | 0.124 | -0.045 | 0.005 | 0.001 | 0.056 | 0.007 |
| No (cat is restricted in a single room) | -0.452 | 0.164 | 0.017 | 0.003 | 0.056 | 0.007 |
| **Outdoor access** |  |  |  |  |  |  |
| Yes | 0.303 | 0.109 | 0.028 | 0.005 | 0.353 | 0.045 |
| No | -1.165 | -0.418 | 0.109 | 0.017 | 0.353 | 0.045 |
| **Access to the street** |  |  |  |  |  |  |
| Always | 0.780 | 0.531 | 0.042 | 0.024 | 0.129 | 0.060 |
| Oftenly | 0.419 | -0.680 | 0.002 | 0.007 | 0.006 | 0.015 |
| Occasionally | 0.708 | -0.709 | 0.024 | 0.029 | 0.069 | 0.069 |
| Never | -0.350 | 0.021 | 0.032 | 0.000 | 0.251 | 0.001 |
| **Visual access to street** |  |  |  |  |  |  |
| Yes | 0.012 | -0.131 | 0.000 | 0.007 | 0.001 | 0.089 |
| No | -0.060 | 0.679 | 0.000 | 0.036 | 0.001 | 0.089 |
| **Access to elevated areas** |  |  |  |  |  |  |
| Yes (in shelves. tables or others) | -0.082 | 0.090 | 0.002 | 0.003 | 0.032 | 0.040 |
| No | 0.397 | -0.440 | 0.010 | 0.016 | 0.032 | 0.040 |
| **Access to cat toys** |  |  |  |  |  |  |
| Yes | 0.004 | -0.372 | 0.000 | 0.055 | 0.000 | 0.674 |
| No | -0.021 | 1.812 | 0.000 | 0.270 | 0.000 | 0.674 |
| **Play with toys (cat toys or objects)** |  |  |  |  |  |  |
| Yes | -0.191 | -0.375 | 0.007 | 0.034 | 0.037 | 0.144 |
| No | 0.739 | -0.149 | 0.027 | 0.001 | 0.079 | 0.003 |
| Only when stimulated | 0.072 | -0.281 | 0.000 | 0.009 | 0.002 | 0.025 |
| Do not have access to toys | -0.108 | 2.203 | 0.001 | 0.294 | 0.002 | 0.697 |
| **Left alone in the house (frequency)** |  |  |  |  |  |  |
| 5 to 7 times per week | -0.656 | 0.049 | 0.082 | 0.001 | 0.411 | 0.002 |
| 1 to 4 times per week | -0.248 | 0.211 | 0.004 | 0.004 | 0.013 | 0.010 |
| Occasionally (less than once a week) | 0.841 | -0.518 | 0.062 | 0.029 | 0.204 | 0.078 |
| Never | 1.639 | 0.504 | 0.113 | 0.013 | 0.324 | 0.031 |
| **Left alone in the house (duration)** |  |  |  |  |  |  |
| < 2 hours / day | 0.921 | -1.172 | 0.037 | 0.074 | 0.107 | 0.173 |
| 2 to 6 hours / day | -0.395 | 0.035 | 0.023 | 0.000 | 0.093 | 0.001 |
| > 6 hours / day | -0.420 | 0.209 | 0.027 | 0.008 | 0.111 | 0.027 |
| Not left alone or do not know | 1.584 | 0.289 | 0.128 | 0.005 | 0.375 | 0.013 |
| **Other animals in the house** |  |  |  |  |  |  |
| Yes | 0.189 | 0.104 | 0.012 | 0.004 | 0.180 | 0.054 |
| No | -0.951 | -0.523 | 0.059 | 0.022 | 0.180 | 0.054 |
| **Change with unfamiliar person** |  |  |  |  |  |  |
| Yes | 0.133 | -0.045 | 0.004 | 0.001 | 0.021 | 0.002 |
| No | -0.158 | 0.053 | 0.004 | 0.001 | 0.021 | 0.002 |
